# High content image analysis for human H4 neuroglioma cells exposed to CuO nanoparticles

**DOI:** 10.1186/1472-6750-7-66

**Published:** 2007-10-09

**Authors:** Fuhai Li, Xiaobo Zhou, Jinmin Zhu, Jinwen Ma, Xudong Huang, Stephen TC Wong

**Affiliations:** 1 The Center for Biomedical Informatics, The Methodist Hospital Research Institute, Weill Cornell Medical College, Houston, TX 77030, USA; 2 Department of Information Science, School of Mathematical Sciences, and LMAM, Peking University, Beijing 100871, China; 3 Research Division, Department of Radiology, The Methodist Hospital, Weill Cornell Medical College, Houston, TX 77030, USA; 4 Functional and Molecular Imaging Center, Brigham and Women's Hospital, Harvard Medical School, Boston, MA 02115, USA; 5 Neurochemistry Laboratory, Department of Psychiatry and Genetics and Aging Research Unit, Massachusetts General Hospital and Harvard Medical School, Boston, MA 02114, USA

## Abstract

**Background:**

High content screening (HCS)-based image analysis is becoming an important and widely used research tool. Capitalizing this technology, ample cellular information can be extracted from the high content cellular images. In this study, an automated, reliable and quantitative cellular image analysis system developed in house has been employed to quantify the toxic responses of human H4 neuroglioma cells exposed to metal oxide nanoparticles. This system has been proved to be an essential tool in our study.

**Results:**

The cellular images of H4 neuroglioma cells exposed to different concentrations of CuO nanoparticles were sampled using IN Cell Analyzer 1000. A fully automated cellular image analysis system has been developed to perform the image analysis for cell viability. A multiple adaptive thresholding method was used to classify the pixels of the nuclei image into three classes: bright nuclei, dark nuclei, and background. During the development of our image analysis methodology, we have achieved the followings: (1) The Gaussian filtering with proper scale has been applied to the cellular images for generation of a local intensity maximum inside each nucleus; (2) a novel local intensity maxima detection method based on the gradient vector field has been established; and (3) a statistical model based splitting method was proposed to overcome the under segmentation problem. Computational results indicate that 95.9% nuclei can be detected and segmented correctly by the proposed image analysis system.

**Conclusion:**

The proposed automated image analysis system can effectively segment the images of human H4 neuroglioma cells exposed to CuO nanoparticles. The computational results confirmed our biological finding that human H4 neuroglioma cells had a dose-dependent toxic response to the insult of CuO nanoparticles.

## Background

A precise determination of cell death model is essential for biomedical researches as cell death pathways are intimately associated with normal physiology and disease-related pathogenesis. The widely used colormetric cytotoxicity assays such as lactate dehydrogenase (LDH) release, MTT [3-(4,5-dimethylthiazol-2-yl)-2,5-diphenyltetrazolium bromide]/MTS [3-(4,5-dimethylthiazol-2-yl)-5-(3-carboxymethoxyphenyl)-2-(4-sulfophenyl)-2H-tetrazolium, inner salt] based assays, etc., can only evaluate the viability of cell ensemble. Thus there is a strong demand for sensitive, quantitative, reliable and automated methods for the accurate assessment of cellular proliferation status with *high contents *of cellular information. As a modern drug discovery tool, high content screening (HCS) [1] using automated fluorescence microscope is becoming an important and widely used research tool to assist researchers understanding complex cellular processes in disease pathogenesis, drug target validation and drug lead identification [2,3]. Using the HCS technology, abundant spatial and temporal morphologic information can be extracted from the cellular images, and the information can be used to determine whether a potential drug affects the functions of proteins or genes involved in a disease process. However, it has been a challenge to perform quantitative analysis of the complex cellular images, and this significantly restricts the potential of HCS in drug discovery [2]. Thus, the availability of fully automated cellular image analysis systems is critical to the success of HCS.

**Figure 1:**
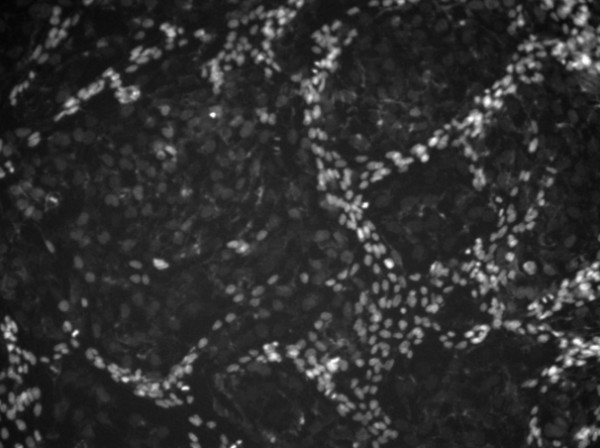
A representative nuclei image acquired in this study.

The fluorescent images of human H4 neuroglioma cells exposed to different concentrations of CuO nanoparticles were collected by a high content fluorescence microscope – IN Cell Analyzer 1000. Using these cellular images, investigators can investigate the influences of the CuO nanoparticles to the cell viability and determine cell death mode by analyzing the percentage of dead/live cells. However, there are thousands of cellular images generated in one experiment, and thousands of cells appear in each image. It is impractical to count and quantify the cells manually. Therefore, a fully automated and robust cellular image analysis system is needed urgently. For a fully automated cellular images analysis system, the detection and segmentation of nuclei are the two essential components [4]. The problems of nuclei segmentation originate from uneven illumination, artifacts, nuclei clustering and low intensity contrast between the nuclei and the background [5]. As seen in Figure 1, nuclei are of different sizes, intensities and shapes, which pose a problem in segmentation. Secondly, the intensity contrast of dark nuclei is very low, and the bright nuclei are found to cluster together. The problem is further aggravated due to the presence of high-intensity noises in the dark nuclei region. Although some methods for fluorescent cellular image detection and segmentation have been proposed, a general purpose system that can perform the detection and segmentation tasks for all kinds of fluorescence microscopy images without any fine tuning is still not available. This has motivated us to design a novel system to serve the purpose.

In [6,7] some nuclei segmentation methods were proposed. They combined the intensity gradient information with the shape information to separate the clustered nuclei by using a statistical model to merge the fragments of nuclei. Since the bright nuclei cluster together heavily, and the dark nuclei cannot be accurately separated from the background, these methods tend to fail because the shape information is not accurate. In addition, edge based segmentation methods will fail due to the noisy and discontinuous edges [8]. Thresholding methods cannot separate the clustered nuclei [9]. Moreover, the contours' initialization of the snake and level set methods is much more challenging work [10-12].

**Figure 2:**
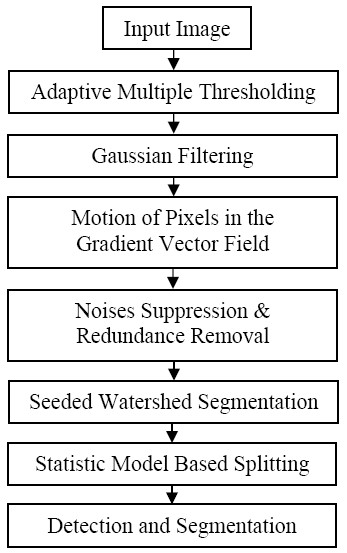
The overview flowchart of the proposed image analysis system.

The goal of the present work is to develop an automated cellular image analysis system for quantitative analysis of viability of H4 neuroglioma cells exposed to CuO nanoparticles. Figure 2 provides a flowchart of the proposed system. We first used a background correction method [13,14] as a multiple adaptive thresholding technique to classify the nuclei image into three classes: bright nuclei, dark nuclei and background. Then we implemented a nuclei detection method based on the Gaussian filtering and gradient vector field [12] followed by the seeded watershed [15,16] based region-growing algorithm to segment the clustered nuclei. Finally we proposed a statistical model based splitting method to reduce the under-segmentation problem.

## Results and discussion

### Materials

Human H4 neuroglioma cells purchased from the ATCC (Manassas, VA) were seeded into 96-well cell culture plates and cultured in Dulbecco's modified Eagle medium (DMEM) supplemented with 10% fetal bovine serum, 1% penicillin-streptomycin solution (Sigma Chemical Co., St. Louis). The cells were incubated for 48 hours under the cell culture conditions (95% O_2_, 5% CO_2_, 85% humidity, 37°C), together with CuO nanoparticles at a concentration range of 0.01–100 *μ*M. Then a live/dead assay kit (Molecular probes/Invitrogen) for cell viability was applied to the cells according to the manufacturer's instruction. In brief, the cells were cultured at 37°C for 30 min, with ethidium homodimer-1 (3 *μ*M, for dead cells), and Hoechst dye (16 *μ*M, for nuclear staining) in each well. High-content cellular fluorescence images were acquired using an automatic fluorescence microscope – IN Cell Analyzer 1000 (GE Healthcare). The objective magnification is 10×. Numerical aperture is 0.45, pixel width is 0.645 *μm *and pixel height is 0.645 *μm *for all the images taken. The size of each image is 1040 × 1392 pixels.

### Choice of parameters

Table 1 gives the values of various parameters used in the proposed method. The pixel classification parameters *c*_*b *_and *c*_*d *_are used to classify pixels of a cellular image into three classes: bright nuclei, dark nuclei and background. These two parameters depend on the intensity contrast between the nuclei and background, and we set their values as 3 and 0.3 empirically for all images in this study. Experimental results show that the proposed nuclei detection method is robust for the two parameters, as seen in Table 2. The rational is that regardless a few background pixels are classified as nuclei or vice versa, the number of pixels converging at the local maxima inside the nuclei will not be affected significantly. Therefore, the detection results will not be altered by a significant amount. The local noise suppression radii, *r*_*d *_and *r*_*b *_are used to suppress redundant central points appeared in the same nuclei, and their values are set to be the minimum radiuses of the bright and dark nuclei respectively. The Gaussian filtering is employed to suppress the noises and generate unique local intensity maximum inside each nucleus. The noisy central points are suppressed by two thresholds: *T*_*b *_and *T*_*d*_. The minimum PDF value of the training nuclei is used as the threshold, *T*_*pdf*_. The nuclei with PDF values less than *T*_*pdf*_, are considered to be under-segmented and are processed in the splitting step. To test the robustness of the proposed method affected by the variation of parameters: *c*_*b*_, *c*_*d*_, *σ*_*b *_and *σ*_*d*_, the image in Figure 1 was selected as a testing example, and the detailed results are given in Table 2.

**Table 1:** Values and description of the parameters used in the proposed method

Parameter	Value	Description
*c*_*b*_	3	Threshold for bright nuclei binarization
*c*_*d*_	0.3	Threshold for dark nuclei binarization
*σ*_*b*_	2.5	Sigma of Gaussian filtering for bright nuclei
*σ*_*d*_	5	Sigma of Gaussian filtering for dark nuclei
*T*_*b*_	50 pixels	Threshold of central point detection of bright nuclei
*T*_*d*_	100 pixels	Threshold of central point detection of dark nuclei
*r*_*b*_	13Db MathType@MTEF@5@5@+=feaafiart1ev1aqatCvAUfKttLearuWrP9MDH5MBPbIqV92AaeXatLxBI9gBaebbnrfifHhDYfgasaacH8akY=wiFfYdH8Gipec8Eeeu0xXdbba9frFj0=OqFfea0dXdd9vqai=hGuQ8kuc9pgc9s8qqaq=dirpe0xb9q8qiLsFr0=vr0=vr0dc8meaabaqaciaacaGaaeqabaqabeGadaaakeaadaWcaaqaaiabigdaXaqaaiabiodaZaaacqWGebardaWgaaWcbaGaemOyaigabeaaaaa@312B@	Radius of local suppression of bright nuclei; *D*_*b *_is the diameter of bright nuclei (*D*_*b *_≈ 9.675 micron).
*r*_*d*_	13Dd MathType@MTEF@5@5@+=feaafiart1ev1aqatCvAUfKttLearuWrP9MDH5MBPbIqV92AaeXatLxBI9gBaebbnrfifHhDYfgasaacH8akY=wiFfYdH8Gipec8Eeeu0xXdbba9frFj0=OqFfea0dXdd9vqai=hGuQ8kuc9pgc9s8qqaq=dirpe0xb9q8qiLsFr0=vr0=vr0dc8meaabaqaciaacaGaaeqabaqabeGadaaakeaadaWcaaqaaiabigdaXaqaaiabiodaZaaacqWGebardaWgaaWcbaGaemizaqgabeaaaaa@312F@	Radius of local suppression of bright nuclei; *D*_*d *_is the diameter of dark nuclei (*D*_*d *_≈ 19.35 micron).
*T*_*pdf*_	*e*^-41^	Threshold of the PDF score

**Table 2:** Robustness test of the parameters: *c*_*b*_, *c*_*d*_, *σ*_*b *_and *σ*_*d*_

*c*_*b*_	2.0	2.5	3	3.5	4
# of detected nuclei	869	864	859	848	833
*c*_*d*_	0.2	0.25	0.3	0.35	0.4
# of detected nuclei	858	862	859	850	835
*σ*_*b*_	1.5	2.0	2.5	3.0	3.5
# of detected nuclei	859	856	859	859	852
*σ*_*d*_	4.0	4.5	5.0	5.5	6.0
# of detected nuclei	852	853	859	857	856

### Validation and comparison of segmentation

**Figure 3:**
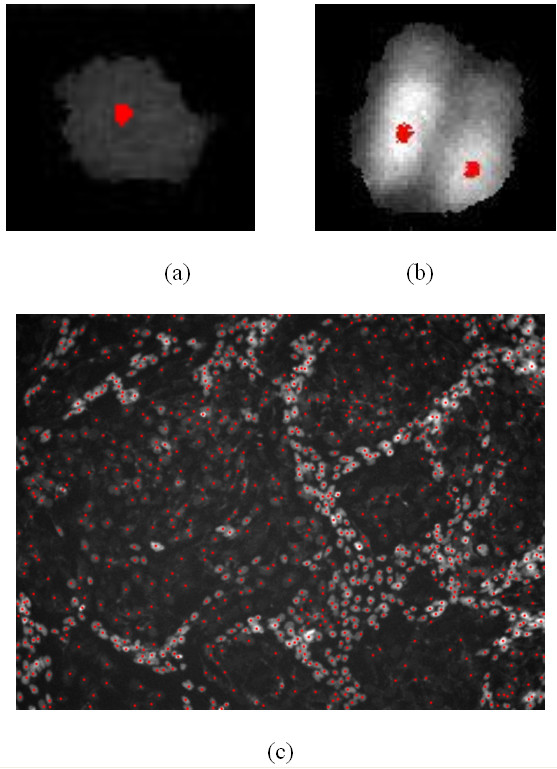
**Results of nuclei detection**. a, b: The detection results of Figure 9-(a) and (d). c: The detection result of Figure 1.

**Figure 4:**
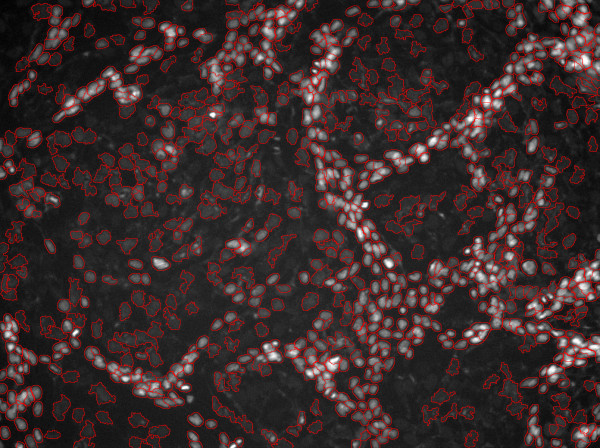
The final segmentation results of Figure 1.

To evaluate the accuracy of the proposed nuclei segmentation method, we randomly picked up ten nuclei images as the testing data set. Figure 3-(c) and Figure 4 show the representative detection and segmentation results respectively. Four possible segmentation errors were considered: over-segmentation, under-segmentation, false negative and false positive (noises). The false positive rate (FPR) and the false negative rate (FNR) are defined as follows:

FPR=#offalsenuclei#oftotalnucleiandFNR=#ofmissednuclei#oftotalnuclei
 MathType@MTEF@5@5@+=feaafiart1ev1aaatCvAUfKttLearuWrP9MDH5MBPbIqV92AaeXatLxBI9gBaebbnrfifHhDYfgasaacH8akY=wiFfYdH8Gipec8Eeeu0xXdbba9frFj0=OqFfea0dXdd9vqai=hGuQ8kuc9pgc9s8qqaq=dirpe0xb9q8qiLsFr0=vr0=vr0dc8meaabaqaciaacaGaaeqabaqabeGadaaakeaafaqabeqadaaabaGaemOrayKaemiuaaLaemOuaiLaeyypa0ZaaSaaaeaacqGGJaWiiiaacqWFGaaicqWGVbWBcqWGMbGzcqWFGaaicqWGMbGzcqWGHbqycqWGSbaBcqWGZbWCcqWGLbqzcqWFGaaicqWGUbGBcqWG1bqDcqWGJbWycqWGSbaBcqWGLbqzcqWGPbqAaeaacqGGJaWicqWFGaaicqWGVbWBcqWGMbGzcqWFGaaicqWG0baDcqWGVbWBcqWG0baDcqWGHbqycqWGSbaBcqWFGaaicqWGUbGBcqWG1bqDcqWGJbWycqWGSbaBcqWGLbqzcqWGPbqAcqWFGaaiaaaabaGaeeyyaeMaeeOBa4MaeeizaqgabaGaemOrayKaemOta4KaemOuaiLaeyypa0ZaaSaaaeaacqGGJaWicqWFGaaicqWGVbWBcqWGMbGzcqWFGaaicqWGTbqBcqWGPbqAcqWGZbWCcqWGZbWCcqWGLbqzcqWGKbazcqWFGaaicqWGUbGBcqWG1bqDcqWGJbWycqWGSbaBcqWGLbqzcqWGPbqAaeaacqGGJaWicqWFGaaicqWGVbWBcqWGMbGzcqWFGaaicqWG0baDcqWGVbWBcqWG0baDcqWGHbqycqWGSbaBcqWFGaaicqWGUbGBcqWG1bqDcqWGJbWycqWGSbaBcqWGLbqzcqWGPbqAcqWFGaaiaaaaaaaa@9071@

Table 3 provides the detailed results of nuclei segmentations. On an average, 95.9% of the nuclei were accurately detected, 0.8% of the nuclei were under-segmented and 2.6% of the nuclei were over-segmented. The FNR and FPR were found to be 0.7% and 5.7% respectively. All the images were processed with the fixed parameters.

**Table 3:** Validation of the proposed method on ten randomly selected nuclei images

Image	# of nuclei (manual counted)	# of nuclei (correctly – segmented)	# of nuclei (over – segmented)	# of nuclei (under – segmented)	# of nuclei (missed)	# of nuclei (noises)
1	858	831 (96.8%)	10 (1.2%)	2 (0.2%)	15 (1.7%)	42 (4.9%)
2	1082	1030 (95.1%)	30 (2.8%)	15 (1.4%)	7 (0.6%)	40 (3.7%)
3	671	653 (97.3%)	12 (1.8%)	5 (0.7%)	1 (0.1%)	41 (6.1%)
4	900	867 (96.3%)	21 (2.3%)	7 (0.8%)	5 (0.6%)	55 (6.1%)
5	723	700 (96.8%)	17 (2.4%)	3 (0.4%)	3 (0.4%)	41 (5.7%)
6	750	712 (94.9%)	26 (3.5%)	5 (0.7%)	7 (0.9%)	54 (7.2%)
7	836	801 (95.8%)	23 (2.8%)	6 (0.7%)	6 (0.7%)	41 (4.9%)
8	720	679 (94.3%)	34 (4.7%)	4 (0.6%)	3 (0.4%)	43 (6.0%)
9	852	811 (95.2%)	20 (2.3%)	8 (0.9%)	13 (1.5%)	53 (6.2%)
10	773	746 (96.5%)	16 (2.1%)	7 (0.9%)	4 (0.5%)	55 (7.1%)
Avg.	817	783 (95.9%)	21 (2.6%)	6 (0.7%)	6 (0.8%)	47 (5.7%)

To further evaluate the effectiveness of proposed method, we compared the segmentation results of the proposed protocol with the CellProfiler [17], which is free available software and based on watershed method. We compared the two methods using: correctly segmented nuclei, FNR and FPR. As indicated in Table 4, the proposed method outperforms the watershed method. Both the FNR and FPR values of the watershed method are much higher than that of the proposed method. The reason is that watershed algorithm missed some dark nuclei, and under-segmented the bright nuclei clusters. The high FPR values of the watershed method were caused by the fluorescent noises.

**Table 4:** Comparison of segmentation results: Watershed vs. the proposed method

Image	Correctly segmented (%)		FNR		FPR	
	
	Proposed method	Watershed method	Proposed method	Watershed method	Proposed method	Watershed method
1	96.8%	80.2%	2.0%	22.1%	6.1%	10.6%
2	95.1%	82.3%	2.1%	16.5%	6.5%	10.3%
3	96.3%	84.3%	0.9%	16.3%	7.9%	8.1%
4	95.2%	85.2%	1.4%	12.4%	8.4%	14.0%
5	95.8%	86.1%	0.9%	13.3%	8.0%	11.0%
Avg.	95.84%	83.62%	1.46%	16.12%	7.38%	10.80%

**Figure 5:**
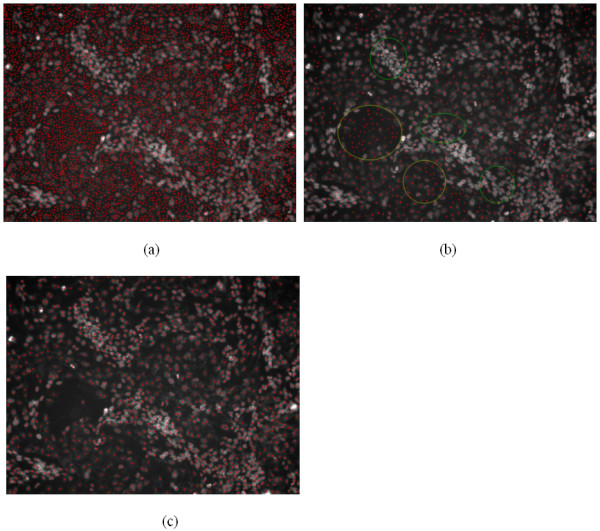
**Comparison of detection results: the ITCN vs. proposed method**. a: The detection result of ITCN with setting the cell diameter parameter as 9.675 micron. b: The detection result of ITCN with setting the cell diameter parameter as 19.35 micron. c: The detection result of the proposed method.

We also tested the cell counting tool in ImageJ – ITCN. This tool was developed by Thomas Kuo and Jiyun Byun (Center for bio-image Informatics). The ITCN uses Laplacian of Gaussian (LOG) filtering as the nuclei detector. There is a parameter, i.e. diameter of a cell, in ITCN, and we tested the tool with two different diameter values of the cell: 9.675 micron (the diameter of the bright nuclei) and 19.35 micron (the diameter of the dark nuclei). The results are shown in Figure 5-(a) and 5-(b) respectively. As seen in Figure 5-(a), there are too many noises (false positives) and over-detection because we used the smaller diameter (9.675 micron). In Figure 5-(b), there are some bright nuclei are under-segmented (see the red circle) when we use larger diameter (19.35 micron). In addition, many noisy points still exist (see the yellow circle). In conclusion, the ITCN results are very sensitive to the initial values of the diameter of cell and the intensity noises. It works well if the cells or nuclei have similar size, intensity and round shape. However, in our study, the two kinds of nuclei have different size, intensity and shape. The bright nuclei clustered together, and the contrast of the dark nuclei is very low. These difficulties limit the performance of ITCN. The detection result of the proposed method is provided in Figure 5-(c).

### Analysis of cell death induced by CuO nanoparticles

**Figure 6:**
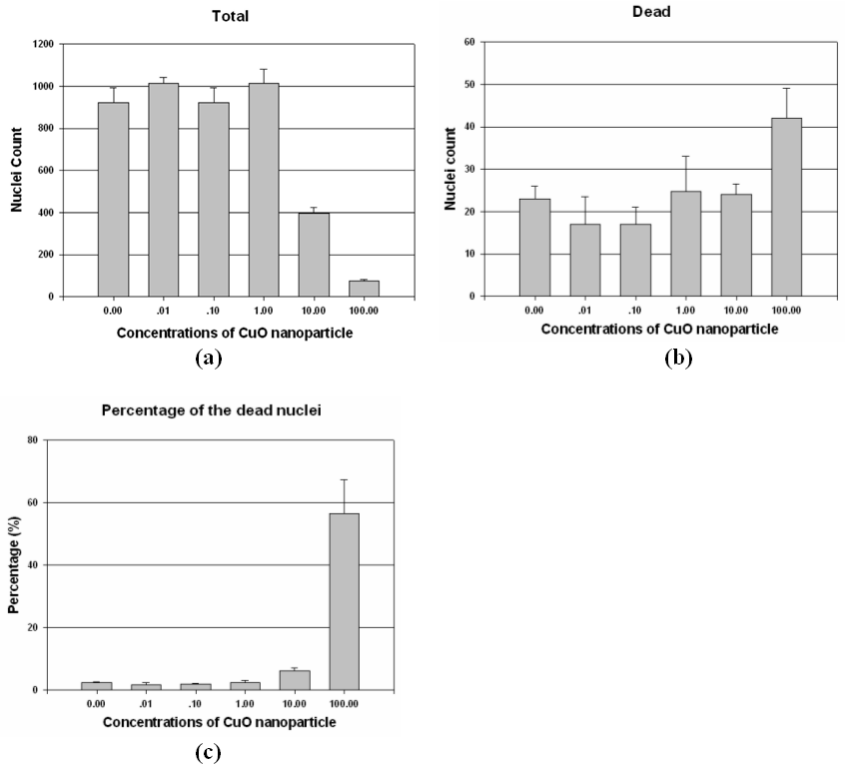
**Block plot of the numbers of the detected nuclei treated with six different concentrations of CuO nanoparticles**. a: Block plot of the numbers of total nuclei. b: Block plot of the numbers of the dead nuclei. c: Block plot of the percentages of the dead nuclei.

In this study, we applied the proposed system to analyze the toxic response of the human H4 cells exposed to the CuO nanoparticles. We treated the human H4 neuroglioma cells with five different concentrations of CuO nanoparticles: 0.01, 0.1, 1, 10, and 100 *μ*M for 48 hours. We used two fluorescence dyes, Hoechst 33258 and ethidium homodimer-1(EthD-1), for staining total cells and dead cells (both from Molecular Probes, Invitrogen), respectively, following the vendor suggested protocols. The blue fluorescent Hoechst dye (ex/em ~350 nm/~460 nm) are cell permeable nucleic acid stains that have multiple applications, including determination of cell number. The fluorescence of the dye is very sensitive to DNA conformation in both live and dead cells. EthD-1, however, enters cells only with damaged membranes and undergoes a 40-fold enhancement of fluorescence upon binding to nucleic acids, thereby producing a bright red fluorescence in dead cells (ex/em ~495 nm/~635 nm). Thus, entry of EthD-1 into living cells with intact plasma membrane is prohibited. The dead and total cells in each well were stained for 2 hours by EthD-1 (3 *μ*M) and Hoechst dye (16 *μ*M), respectively. We acquired the cellular images using the IN Cell Analyzer 1000, an automatic fluorescence microscope after the staining step. The toxic effects of the CuO nanopaticles upon the human H4 neuroglioma cells can be quantified by determining the percentages of dead/total cells treated with different concentrations of CuO nanoparticles. Thus we only need to count the number of total and dead cells using our automatic image analysis system. Figure 6-(a), (b) and 6-(c) give the mean and standard deviation of total cells, dead cells, and percentage of dead/total cells in the untreated and the five different concentrations of CuO nanoparticles treated wells. To determine the statistical significance, we also performed the student's t-tests for cell death ratios (percentage of dead/total cell) between the CuO nanoparticle treated and untreated human H4 neuroglioma cells. Table 5 gives the p-values of these t-tests. From Figure 6 and Table 5, we conclude that the increases of cell death ratio is statistically significant in H4 cells treated by >10 *μ*M of CuO nanoparticles (significance level: *α *= 0.01). The computational results are consistent with our biological finding that human H4 neuroglioma cells have a dose-dependent toxic response to the insult of CuO nanoparticles. Interestingly, Benson JM., et al. [18] reported that *in vitro *cytotoxicity of the Ni-CuO compounds to pulmonary alveolar macrophages is correlated to their increased Cu content and decreased Ni content. As such, our results, which are in good agreement with Benson et al., indicate CuO nanoparticles are cytotoxic to human brain cells.

**Table 5:** P-values of the T tests for H4 cell death rate comparison: the CuO nanoparticles treated vs. untreated

Concentration of CuO nanoparticle (*μ*M)	0.01	0.1	1	10	100
p-value	0.041	0.018	0.271	0.009	0.0008

## Conclusion

Herein we present a fully automated cellular image analysis system for quantitative analysis of the viability of human H4 neuroglioma cells exposed to CuO nanoparticles with different concentrations (0.01 – 100 *μ*M). A multiple thresholding method was used to classify nuclei image into three classes: bright nuclei, dark nuclei, and background, based on the background correction algorithm. Following this, a method for fining local image intensity maxima using the Gaussian filtering and gradient vector field was developed to detect the nuclei. A statistical model based splitting method was proposed to reduce the under segmentation problem. The experimental results show that 95.9% nuclei are segmented correctly using the proposed image analysis protocol. Its application on our experimental data sets further indicates that the human H4 neuroglioma cells have a concentration-dependent toxic response to the insult of CuO nanoparticles.

## Methods

### Image pre-processing and pixel classification

No imaging system is perfect, and it is imperative to perform pre-processing to remove the effects of noises, artifacts, uneven illumination, and striped patterns [6,7,13,14] that degrade image quality. To remove the noises and other artifacts without blurring the edges, the median filtering [6,7] was applied. For uneven illumination and striped patterns, a data driven background correction algorithm [13,14] was employed to correct the degeneration of the images. The algorithm makes use of the cubic B-splines which have good features, such as continuouity and smoothness, to estimate the background iteratively, and the convergence of this algorithm is fast. Image pre-processing produced images with improved quality.

In this study, the pixel classification means to classify each pixel into the one of three classes: background, dark nuclei and bright nuclei. There are two reasons for doing pixel classification. First, separating the nuclei pixels from the background can reduce the influence of the background in following dark nuclei and bright nuclei detection. Secondly, two kinds of nuclei: bright nuclei and dark nuclei displayed different features in the image, as shown in Figure 1. The bright nuclei, which have high intensity, rice shape, and smaller size, form a tight cluster. The dark nuclei, which have low intensity, round shape and larger size, are scattered. Hence it is reasonable to analyze the bright and dark nuclei separately due to their different attributes.

To achieve pixel classification, we employed the background correction algorithm [13,14] as a multiple adaptive thresholding method. The basic idea of this method is straightforward. We can visually separate the nuclei from the background into different classes due to the discontinuity of intensity between the background and the two kinds of nuclei. Based on this fact, we can classify one pixel into one of the three classes based on its intensity difference between the real image and the estimated background image obtained by the background correction algorithm. Mathematically the multiple adaptive thresholding method can be written as:

*Q*(*x*, *y*, *c*) = *q*(*I*(*x*, *y*) - *B*(*x*, *y*) - c*σ_*B*_)

where *I*(*x*, *y*) is the image function; *B*(*x*, *y*) is the estimated background function with cubic B-spline; *c *is a control parameter and *σ*_*B *_is the standard deviation of the gray level of estimated background. *q*(·)is an indicator function:

q(z)={0,z<0;1,z≥0.
 MathType@MTEF@5@5@+=feaafiart1ev1aaatCvAUfKttLearuWrP9MDH5MBPbIqV92AaeXatLxBI9gBaebbnrfifHhDYfgasaacH8akY=wiFfYdH8Gipec8Eeeu0xXdbba9frFj0=OqFfea0dXdd9vqai=hGuQ8kuc9pgc9s8qqaq=dirpe0xb9q8qiLsFr0=vr0=vr0dc8meaabaqaciaacaGaaeqabaqabeGadaaakeaacqWGXbqCcqGGOaakcqWG6bGEcqGGPaqkcqGH9aqpdaGabeqaauaabaqaciaaaeaacqaIWaamcqGGSaalaeaacqWG6bGEcqGH8aapcqaIWaamcqGG7aWoaeaacqaIXaqmcqGGSaalaeaacqWG6bGEcqGHLjYScqaIWaamcqGGUaGlaaaacaGL7baaaaa@4096@

**Figure 7:**
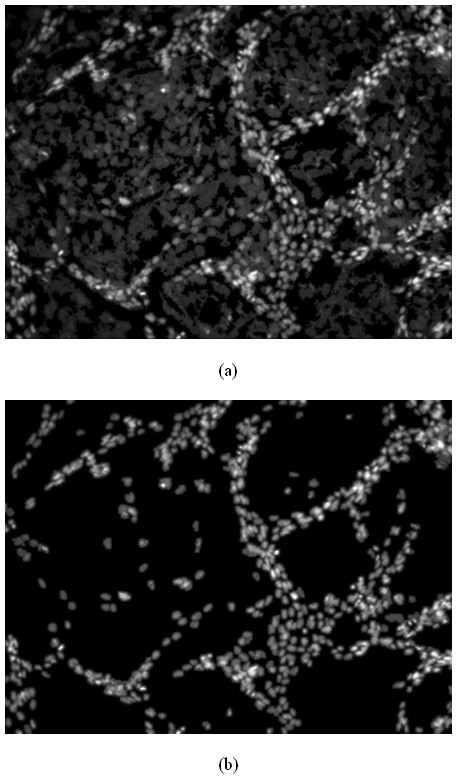
**Binarization results of Figure 1**. a: The first threshold separates the nuclei from the background. b: The second threshold separates the bright nuclei from the dark nuclei.

If *Q*(*x*, *y*, *c*) = 1, image pixel (*x*, *y*) is classified as the object, otherwise, the image pixel (*x*, *y*) is classified as background. In Equation (1), it can be seen that the result of pixel classification depends directly on the parameter *c*: the higher the value of the parameter *c *is, the more pixels are separated into the background. To classify the pixels of nuclei image into three classes, we used the proposed adaptive threshold method as follows: first, we used a lower value, *c*_*d*_, to separate both of the bright and dark nuclei from the background, as seen in Figure 7-(a). Secondly we chose a higher threshold, *c*_*b*_, to separate the bright nuclei from the dark nuclei, as seen in Figure 7-(b). The classification process can be mathematically written as:

Cd⊕b(x,y)={0,Q(x,y,cd)=0;I(x,y),Q(x,y,cd)=1.
 MathType@MTEF@5@5@+=feaafiart1ev1aaatCvAUfKttLearuWrP9MDH5MBPbIqV92AaeXatLxBI9gBaebbnrfifHhDYfgasaacH8akY=wiFfYdH8Gipec8Eeeu0xXdbba9frFj0=OqFfea0dXdd9vqai=hGuQ8kuc9pgc9s8qqaq=dirpe0xb9q8qiLsFr0=vr0=vr0dc8meaabaqaciaacaGaaeqabaqabeGadaaakeaacqWGdbWqdaWgaaWcbaGaemizaqMaeyyLIuSaemOyaigabeaakiabcIcaOiabdIha4jabcYcaSiabdMha5jabcMcaPiabg2da9maaceqabaqbaeaabiGaaaqaaiabicdaWiabcYcaSaqaaiabdgfarjabcIcaOiabdIha4jabcYcaSiabdMha5jabcYcaSiabdogaJnaaBaaaleaacqWGKbazaeqaaOGaeiykaKIaeyypa0JaeGimaaJaei4oaSdabaGaemysaKKaeiikaGIaemiEaGNaeiilaWIaemyEaKNaeiykaKIaeeilaWcabaGaemyuaeLaeiikaGIaemiEaGNaeiilaWIaemyEaKNaeiilaWIaem4yam2aaSbaaSqaaiabdsgaKbqabaGccqGGPaqkcqGH9aqpcqaIXaqmcqGGUaGlaaaacaGL7baaaaa@5E35@

Cd(x,y)={0,Q(x,y,cd)=0;I(x,y),Q(x,y,cd)=1 and Q(x,y,cb)=0;0,Q(x,y,cb)=1.
 MathType@MTEF@5@5@+=feaafiart1ev1aaatCvAUfKttLearuWrP9MDH5MBPbIqV92AaeXatLxBI9gBaebbnrfifHhDYfgasaacH8akY=wiFfYdH8Gipec8Eeeu0xXdbba9frFj0=OqFfea0dXdd9vqai=hGuQ8kuc9pgc9s8qqaq=dirpe0xb9q8qiLsFr0=vr0=vr0dc8meaabaqaciaacaGaaeqabaqabeGadaaakeaacqWGdbWqdaWgaaWcbaGaemizaqgabeaakiabcIcaOiabdIha4jabcYcaSiabdMha5jabcMcaPiabg2da9maaceqabaqbaeaabmGaaaqaaiabicdaWiabcYcaSaqaaiabdgfarjabcIcaOiabdIha4jabcYcaSiabdMha5jabcYcaSiabdogaJnaaBaaaleaacqWGKbazaeqaaOGaeiykaKIaeyypa0JaeGimaaJaei4oaSdabaGaemysaKKaeiikaGIaemiEaGNaeiilaWIaemyEaKNaeiykaKIaeiilaWcabaGaemyuaeLaeiikaGIaemiEaGNaeiilaWIaemyEaKNaeiilaWIaem4yam2aaSbaaSqaaiabdsgaKbqabaGccqGGPaqkcqGH9aqpcqaIXaqmcqqGGaaicqWGHbqycqWGUbGBcqWGKbazcqqGGaaicqWGrbqucqGGOaakcqWG4baEcqGGSaalcqWG5bqEcqGGSaalcqWGJbWydaWgaaWcbaGaemOyaigabeaakiabcMcaPiabg2da9iabicdaWiabcUda7aqaaiabicdaWiabcYcaSaqaaiabdgfarjabcIcaOiabdIha4jabcYcaSiabdMha5jabcYcaSiabdogaJnaaBaaaleaacqWGIbGyaeqaaOGaeiykaKIaeyypa0JaeGymaeJaeiOla4caaaGaay5Eaaaaaa@7BEF@

Cb(x,y)={0,Q(x,y,cb)=0;I(x,y),Q(x,y,cb)=1.
 MathType@MTEF@5@5@+=feaafiart1ev1aaatCvAUfKttLearuWrP9MDH5MBPbIqV92AaeXatLxBI9gBaebbnrfifHhDYfgasaacH8akY=wiFfYdH8Gipec8Eeeu0xXdbba9frFj0=OqFfea0dXdd9vqai=hGuQ8kuc9pgc9s8qqaq=dirpe0xb9q8qiLsFr0=vr0=vr0dc8meaabaqaciaacaGaaeqabaqabeGadaaakeaacqWGdbWqdaWgaaWcbaGaemOyaigabeaakiabcIcaOiabdIha4jabcYcaSiabdMha5jabcMcaPiabg2da9maaceqabaqbaeaabiGaaaqaaiabicdaWiabcYcaSaqaaiabdgfarjabcIcaOiabdIha4jabcYcaSiabdMha5jabcYcaSiabdogaJnaaBaaaleaacqWGIbGyaeqaaOGaeiykaKIaeyypa0JaeGimaaJaei4oaSdabaGaemysaKKaeiikaGIaemiEaGNaeiilaWIaemyEaKNaeiykaKIaeeilaWcabaGaemyuaeLaeiikaGIaemiEaGNaeiilaWIaemyEaKNaeiilaWIaem4yam2aaSbaaSqaaiabdkgaIbqabaGccqGGPaqkcqGH9aqpcqaIXaqmcqGGUaGlaaaacaGL7baaaaa@5AD4@

where *C*_*d*⊕*b*_(*x*, *y*), *C*_*d*_(*x*, *y*) and *C*_*b*_(*x*, *y*) denote the classes of nucleus (dark and bright), dark nucleus, and bright nucleus, respectively. The noisy fragments were removed based on the size, and the holes on the nuclei objects were considered as the noisy fragments in the negative image. The two thresholds, *c*_*d *_and *c*_*b*_, were obtained experimentally, and we processed all the nuclei images with the same *c*_*d *_and *c*_*b*_. The parameter selection is discussed in more detail in the 'Choice of parameters' Section. In the following sections, we used the dark nuclei image and bright nuclei image to denote the images which only contain the dark nuclei pixels and the bright nuclei pixels, respectively.

### Nuclei detection

Although the nuclei are separated from the background by the multiple adaptive threshold method, many clustered nuclei are under-segmented. To segment the clustered nuclei, the positions of nuclei need to be detected, which serves as the seed points of the seeded watershed segmentation algorithm. In the following, we propose a nuclei detection method using the Gaussian filtering and gradient vector field.

#### Gaussian filtering

**Figure 8:**
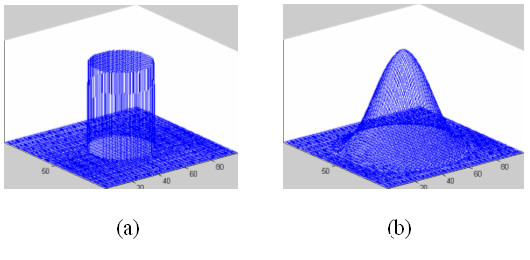
**Illustrations of properties of Gaussian filter with artificial images**. a: Intensity distribution of two dimensional images. b: Intensity distribution of the images after the Gaussian filtering.

**Figure 9:**
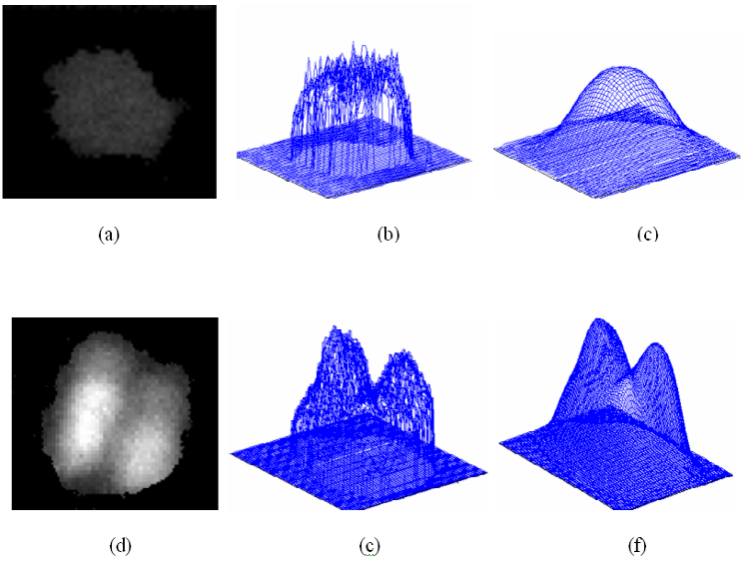
**Illustrations of properties of Gaussian filter with real images**. a, d: Two nuclei images. b, e: The intensity distributions of a and b before Gaussian filtering. c, f: The intensity distributions of the a and d after the Gaussian filtering.

The objective of using Gaussian filtering is to generate a unique local intensity maximum inside each nucleus, which can be used to represent the positions of the nuclei. In what follows, we discuss the two useful properties of Gaussian filtering used in this study. First, suppose a nucleus has the uniform intensity distribution, a unique local maximum inside the nucleus will be generated after Gaussian filtering, as seen in Figure 8. Since the intensity of dark nuclei has approximately uniform intensity distribution, as seen in Figure 9-(a) and 9-(b), we can use the Gaussian filtering to generate a unique local intensity maximum inside dark nuclei, as seen in Figure 9-(c). Secondly, Gaussian filtering is a good smoother and noise suppressor. Since both of the dark and bright nuclei have many noisy local maxima, as seen in Figure 9-(d) and 9-(e), noise reduction is necessary to avoid false detection. Gaussian filtering suppresses these noise local maxima and generates a unique local maximum, as seen in Fig 9-(f). Thus the positions of the nuclei can be represented by the local maxima of the Gaussian filtered image, and the detection of nuclei is reduced to the local intensity maxima detection problem. The attributes of the nuclei, e.g. size and the distribution of intensity, dictate the choice of proper *σ *of Gaussian filtering, so we need to choose different *σ *for the dark nuclei image and the bright nuclei image respectively. The proper value of *σ *can be selected experimentally based on some training images. In current study, the values of *σ *for the bright and dark nuclei were set to be 2.5 and 5.0, respectively. In *Section of 'choice of parameters'*, we discussed the robustness of proposed method for the two parameters.

#### Detection of the central points (nuclei)

We used the central points to denote the local intensity maxima inside nuclei. As discussed above, the problem of nuclei detection has been reduced to the detection of the local intensity maxima, thus the detection of central points means the detection of the nuclei. In this study, we implemented a central point detection method using the gradient vector field of the filtered nuclear images. Mathematically, the gradient vector field of an image is defined as following:

F⇀(x,y)=∂I(x,y)∂xi+∂I(x,y)∂yj
 MathType@MTEF@5@5@+=feaafiart1ev1aaatCvAUfKttLearuWrP9MDH5MBPbIqV92AaeXatLxBI9gBaebbnrfifHhDYfgasaacH8akY=wiFfYdH8Gipec8Eeeu0xXdbba9frFj0=OqFfea0dXdd9vqai=hGuQ8kuc9pgc9s8qqaq=dirpe0xb9q8qiLsFr0=vr0=vr0dc8meaabaqaciaacaGaaeqabaqabeGadaaakeaacuWGgbGrgaGdaiabcIcaOiabdIha4jabcYcaSiabdMha5jabcMcaPiabg2da9maalaaabaGaeyOaIyRaemysaKKaeiikaGIaemiEaGNaeiilaWIaemyEaKNaeiykaKcabaGaeyOaIyRaemiEaGhaaiabdMgaPjabgUcaRmaalaaabaGaeyOaIyRaemysaKKaeiikaGIaemiEaGNaeiilaWIaemyEaKNaeiykaKcabaGaeyOaIyRaemyEaKhaaiabdQgaQbaa@4DEA@

where *I*(*x*, *y*) is an image function. It is well known that, in the electric field, the free negative electrons move along the electric field lines and stop at the positive electrodes. In the gradient vector filed, the gradient vector lines point to the local maxima of the filtered images. If we view the local maxima and the detected nuclei pixels as the positive electrodes and the free negative electrons respectively, by the same analogy, the nuclei pixels of a nucleus will move along the gradient vector lines in the gradient vector field and at last stop at the central point inside the nucleus. Therefore, these central points will be covered by a number of nuclei pixels whereas the non central points have no one pixel stops at them. Based on this fact, we let the detected nuclei pixels move along the gradient vector lines first, and then the central points can be detected by finding the points which are covered by a significant number of pixels. The motion of pixels along with the gradient vector lines can be achieved as follows: given a pixel (*x*_0_, *y*_0_), let it move along the direction of the gradient vector in point (*x*_0_, *y*_0_) to its nearest neighbour (*x*_1_, *y*_1_), and then pixel (*x*_0_, *y*_0_) moves again along the direction of the gradient vector in point (*x*_1_, *y*_1_) to the next nearest point (*x*_2_, *y*_2_). Repeating this process, pixel (*x*_0_, *y*_0_) at last will stop at a local maximum. In these detected central points, some noises and redundant (more than one central points appearing inside a single nucleus) central points exist. To suppress the noises, we removed the central points with convergent pixels less than a certain number, *T*_*b*_, for the bright nuclei central points, and *T*_*d *_for the dark nuclei central points. We applied the following criterion to reduce the redundant central points: if the distance between two central points is less than a threshold, *r*_*b*_, for the bright nuclei central points, and *r*_*d *_for the dark nuclei central points, the one with fewer convergent pixels is removed. Finally the detection results of bright and dark nuclei were pooled together. Figure 3-(a) and 3-(b) show the detection results of Figure 9-(a) and 9-(d). Figure 3-(c) shows the detection result of the Figure 1.

### Statistical model based splitting method for refining the nuclei detection

**Figure 10:**
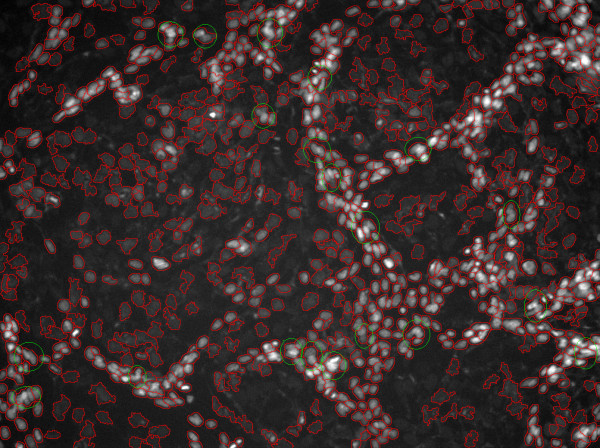
The initial segmentation results of Figure 1.

There is no single detection algorithm can serve as a panacea for the over- and under-detection problem. Although, in the proposed method we reduced the over-detection by thresholding and suppressing the redundant local maxima, there are still some under-detected nuclei especially in the bright nuclei class, as seen the green circles in Figure 10. To address the under-detection issue, we proposed to use the nuclei's shape information to further improve the detection results. Specifically, we first segmented the nuclei images based on the nuclei detection results and seeded watershed algorithm. Then, we used the Gaussian probability density function (PDF) [6,7] to measure the probability of a given segmented nucleus that belongs to a known population which consisted of the well-segmented nuclei, thus the under-segmented nuclei candidates which have lower PDF value can be detected. Finally, all of the detected under-segmented nuclei were sent to a proposed splitting procedure in which the under-segmented nuclei are split under the direction of the PDF values. We describe the proposed method in detail as follows:

#### Nuclei segmentation

Snake model, level-set and seeded watershed methods are a few popular segmentation techniques. However, the snake models need the initial contours near to the true boundaries; the level set method has high computational expenses. Here we employed the seeded watershed based region growing algorithm to segment the nuclei. Figure 10 shows the initial segmented result of Figure 1.

#### Gaussian Probability density function (PDF) model

Before splitting the under-segmented nuclei, we need to detect them first. We reasoned that there should be measurable differences between under-segmented nuclei and well-segmented nuclei. Usually the statistic PDF model combined with a set of features is widely used [6,7] to distinguish the under-segmented nuclei from well-segmented nuclei. The PDF model measures the probability of a given nucleus belonging to a known population which consists of the well-segmented nuclei in a given multiple attributes space. The form of PDF model is given as:

P(x)=1(2π)m/2|Σx|1/2exp⁡{−12(x−x⇀)TΣx−1(x−x⇀)}
 MathType@MTEF@5@5@+=feaafiart1ev1aaatCvAUfKttLearuWrP9MDH5MBPbIqV92AaeXatLxBI9gBaebbnrfifHhDYfgasaacH8akY=wiFfYdH8Gipec8Eeeu0xXdbba9frFj0=OqFfea0dXdd9vqai=hGuQ8kuc9pgc9s8qqaq=dirpe0xb9q8qiLsFr0=vr0=vr0dc8meaabaqaciaacaGaaeqabaqabeGadaaakeaacqWGqbaucqGGOaakcqWH4baEcqGGPaqkcqGH9aqpdaWcaaqaaiabigdaXaqaaiabcIcaOiabikdaYGGaciab=b8aWjabcMcaPmaaCaaaleqabaGaemyBa0Maei4la8IaeGOmaidaaOGaeiiFaWNaeu4Odm1aaSbaaSqaaGqabiab+Hha4bqabaGccqGG8baFdaahaaWcbeqaaiabigdaXiabc+caViabikdaYaaaaaGccyGGLbqzcqGG4baEcqGGWbaCcqGG7bWEcqGHsisldaWcaaqaaiabigdaXaqaaiabikdaYaaacqGGOaakcqWH4baEcqGHsislcuGF4baEgaGdaiabcMcaPmaaCaaaleqabaGaemivaqfaaOGaeu4Odm1aa0baaSqaaiabhIha4bqaaiabgkHiTiabigdaXaaakiabcIcaOiabhIha4jabgkHiTiqb+Hha4zaaoaGaeiykaKIaeiyFa0haaa@5FF5@

where **x **is the *m *dimensional feature vector of a given object; x⇀
 MathType@MTEF@5@5@+=feaafiart1ev1aaatCvAUfKttLearuWrP9MDH5MBPbIqV92AaeXatLxBI9gBaebbnrfifHhDYfgasaacH8akY=wiFfYdH8Gipec8Eeeu0xXdbba9frFj0=OqFfea0dXdd9vqai=hGuQ8kuc9pgc9s8qqaq=dirpe0xb9q8qiLsFr0=vr0=vr0dc8meaabaqaciaacaGaaeqabaqabeGadaaakeaaieqacuWF4baEgaGdaaaa@2E40@ and Σ_**x **_are the mean value and covariance matrix of a known population, respectively; x⇀
 MathType@MTEF@5@5@+=feaafiart1ev1aaatCvAUfKttLearuWrP9MDH5MBPbIqV92AaeXatLxBI9gBaebbnrfifHhDYfgasaacH8akY=wiFfYdH8Gipec8Eeeu0xXdbba9frFj0=OqFfea0dXdd9vqai=hGuQ8kuc9pgc9s8qqaq=dirpe0xb9q8qiLsFr0=vr0=vr0dc8meaabaqaciaacaGaaeqabaqabeGadaaakeaaieqacuWF4baEgaGdaaaa@2E40@ and Σ_**x **_are estimated by the sample mean value and sample covariance matrix of a training data set. The efficiency of PDF model depends on the training data set and the selected features. From the initial segmentation results, we selected 200 well-segmented nuclei as the training data set. Automatic feature selection in a pool of features is a realistic strategy to assemble a good subset of features [19,20]. Since the paucity of the training data set for under-segmented nuclei, we chose nine features empirically, as seen in Table 6.

**Table 6:** Features used in the PDF model

Area	Eccentricity	Solidity
Major Axis Length	Minor Axis Length	Convexity
Standard deviation of intensity	Average Intensity	Roughness

#### Splitting under-segmented nuclei

**Figure 11:**
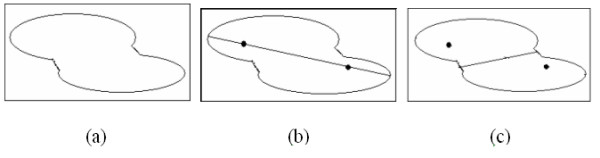
**Illustration of the proposed splitting method**. (a) Overlapped nuclei model. (b) Major axis of the overlapped nuclei and its quarter, three-quarter positions. (c) Separated nuclei.

After measuring the initial segmented nuclei with the PDF model, the under-segmented nuclei often obtain low PDF scores. Only nuclei whose PDF score are lower than a given threshold, *T*_*pdf*_, will be sent to the following splitting step. Wahlby et al. in [14] proposed a splitting method based on the concavities of the overlapped nuclei. However, it is complicated to find the final splitting line from a set of candidates of splitting lines. In this paper, we propose a simple and efficient splitting method, which is intuitively illustrated in Figure 11. Given an under-segmented nucleus, its major axis is extracted first as seen in Figure 11-(a). Following this, two points located in the quarter and three-quarter positions of the major axis are selected as the centers of the overlapped nuclei as shown in Figure 11-(b). Finally, seeded watershed algorithm is applied to segment the overlapped nuclei as indicated in Figure 11-(c).

After obtaining two new nuclei via the splitting step, it is assumed that the PDF scores of the two new generated nuclei should be greater than the original one. Thus the following criterion is established for splitting under-segmented nuclei: if *P*(**x**_*c*_) ⟨ *P*(**x**_*c*2_) and *P*(**x**_*c*_) ⟨ *P*(**x**_*c*2_), we accept the splitting result; otherwise, we reject the splitting result. The new nuclei obtained from the splitting step are measured by the PDF model again, and the nuclei whose PDF values are less than the given threshold, *T*_*pdf*_, are sent to the splitting step again. This process is repeated until no new nucleus is generated. Figure 4 presents the final segmentation result. The software of the proposed system is available, see Additional file 1.

## Competing interests

The author(s) declares that there are no competing interests.

## Authors' contributions

FL participated in the method design, performed the experimental work and drafted the manuscript. XZ guided in the method design and completed the manuscript. JZ acquired the data and performed result analysis. JM guided the method design. XH participated in drafting and revising the manuscript and performed final data analysis. SW guided this study, edited the manuscript and provided research funding. All authors read and approved the final manuscript.

## Supplementary Material

Additional file 1 **The software (matlab codes) package of nuclei detection and segmentation**. It is a .rar file, and the users can use decompress software to open it. In this package, there are the matlab codes, examples and usages of detection and segmentation. The users can test it and validate it. Click here for file

## References

[B1] Perlman Z, Slack M, Feng Y, Mitchison T, Wu L, Altschuler S (2004). Multidimensional drug profiling by automated microscopy. Science.

[B2] Zhou X, Wong S (2006). High content cellular imaging for drug development. IEEE Signal Processing Magazine.

[B3] Zhou X, Wong S (2006). Informatics challenges of high-throughput microscopy. IEEE Signal Processing Magazine.

[B4] Adiga U, Malladi R, Fernandez-Gonzalez R, de Solorzano CO (2006). High-throughput analysis of multispectral images of breast cancer tissue. IEEE Transactions on Image Processing.

[B5] Nattkemper T (2004). Automatic segmentation of digital micrographs: a survey. Medical Informatics.

[B6] Lin G, Adiga U, Olson K, Guzowski J, Barnes C, Roysam B (2003). A hybrid 3-D watershed algorithm incorporating gradient cues and object models for automatic segmentation of nuclei in confocal image stacks. Cytometry A.

[B7] Lin G, Chawla M, Olson K, Guzowski J, Barnes C, Roysam B (2005). Hierarchical, model-based merging of multiple fragments for improved three-dimensional segmentation of nuclei. Cytometry A.

[B8] Garbay C (1986). Image structure representation and processing discussion of some segmentation methods in cytology. IEEE Transactions on Pattern Analysis and Machine Intelligence.

[B9] Kohler R (1981). A Segmentation System Based on Thresholding. Compter Graphics and Image Processing.

[B10] Osher S, Sethian J (1988). Fronts propagating with curvature dependent speed: Algorithms based on the hamilton-jacobi formalism. Journal of Computational Physics.

[B11] Sethian J (1996). Level Set Methods: Evolving interfaces in geometry, fluid mechanics, computer vision, and material science.

[B12] Chen X, Zhou X, Wong S (2006). Automated segmentation, classification, and tracking of cancer cell nuclei in time-lapse microscopy. IEEE Transactions on Biomedical Enineering.

[B13] Lindblad J, Wahlby C, Bengtsson E, Zaltsman A (2004). Image analysis for automatic segmentation of cytoplasms and classification of Rac1 activation. Cytometry A.

[B14] Wahlby C, Lindblad J, Vondrus M, Bengtsson E, Bjorkesten L (2002). Algorithms for cytoplasm segmentation of fluorescence labelled cells. Anal Cell Pathol.

[B15] Beucher S (1992). The watershed transformation applied to image segmentation. Scanning Microscopy International.

[B16] Vincent L, Soille P (1991). Watersheds in digital spaces: an efficient algorithm based on immersion simulations. IEEE Transactions on Pattern Analysis and Machine Intelligence.

[B17] CellProfiler. http://www.cellprofiler.org/.

[B18] Benson JM, Henderson RF, Pickrell JA (1988). Comparative in vitro cytotoxicity of nickel oxides and nickel-copper oxides to rat, mouse, dog pulmonary alveolar macrophages. Journal of toxicology and environmental health.

[B19] Wang J, Zhou X, Li F, Wong S (2006). Classify Cellular Phenotype in High-Throughput Fluorescence Microcopy Images for RNAi Genome-Wide Screening. IEEE/NLM Life Science Systems & Applications Workshop.

[B20] Pudil P, Novovičová J, Kittler J (1994). Floating search methods in feature selection. Pattern Recognition Letters.

